# Efficacy and safety of tramiprosate in Alzheimer’s disease: A systematic review and meta‐analysis

**DOI:** 10.1002/alz.084063

**Published:** 2025-01-03

**Authors:** Ahmad Bouhuwaish

**Affiliations:** ^1^ Faculty of medicine Tobruk University, tobruk, Butnan Libya

## Abstract

**Background:**

Alzheimer’s disease (AD) is a common neurodegenerative disease. Tramiprosate is an amyloid protein (Aß) antagonist. It binds to soluble Aß and prevents conformational transitions that progress to plaque deposition. In this systematic review and meta‐analysis, we aim to evaluate the efficacy and safety of tramiprosate in the treatment of Alzheimer’s disease.

**Method:**

We searched PubMed, Scopus, Web of Science, and Cochrane library on 10/6/2020 and then updated the search on 2/5/2022 for clinical trials that compare between tramiprosate and placebo in Alzheimer’s disease. We pooled data as mean difference (MD) or relative risk (RR) values using Review Manager 5.3 for windows.

**Result:**

Five studies were included in the qualitative analysis and only three of them were eligible for quantitative analysis. There was no significant difference between tramiprosate and placebo in terms of baseline‐endpoint change in Alzheimer’s disease assessment scale ‐ Cognitive subscale Score; neither in DIB 100 mg dose (MD = ‐.08, 95%CI [‐1.82, 1.65], P = 0.09) nor in DIB 150 mg (MD = ‐0.22, 95%CI [‐1.96, 1.51], P = 0.80). Also, there was no significant difference between tramiprosate and placebo in terms of baseline‐endpoint change in Clinical Dementia Rating Scale – Sum of boxes; neither in DIB 100 mg dose (MD = 0.08, 95%CI [‐0.41, 0.57], P = 0.75) nor in DIB 150 mg (MD = 0.20, 95%CI [‐0.29, 0.69], P = 0.42). Regarding safety, there was no significant difference between tramiprosate in terms of DIB 100 mg dose and placebo in side effects except in the incidence of nausea that was significantly higher in the placebo than in tramiprosate groups (RR = 1.41, 95% CI [1.02,1.94], p = 0.04). In terms of DIB 150 mg dose, there was no significant difference between tramiprosate and placebo in the incidence of side effects except in nausea (RR = 1.80, 95% CI [1.32, 2.44], p = 0.0002), vomiting (RR = 1.75, 95% CI [1.18, 2.61], p = 0.006), and decreased weight (RR = 2.28, 95% CI [1.49, 3.49], p = 0.0002) that were significantly higher in placebo than tramiprosate groups.

**Conclusion:**

tramiprosate is not effective in the treatment of AD, although it may have fewer side effects. We recommend other studies to investigate mechanisms other than targeting Aß in the treatment of AD.

